# Apigenin upregulation of CD26/DPPIV on colon epithelial cells requires inhibition of casein kinase 2

**DOI:** 10.1002/fsn3.1823

**Published:** 2020-08-20

**Authors:** Émilie C. Lefort, Bogdan Diaconu, Victoria L. Bentley, Jonathan Blay

**Affiliations:** ^1^ Department of Pathology Dalhousie University Halifax NS Canada; ^2^ School of Pharmacy University of Waterloo Waterloo ON Canada

**Keywords:** 4,5,6,7‐tetrabromobenzotriazole (TBB), 5,6‐dichlorobenzimidazole 1‐β‐D‐ribofuranoside (DRB), 6‐methyl‐1,3,8‐trihydroxyanthraquinone (emodin), apigenin, casein kinase 2, CD26, DPPIV

## Abstract

CD26/DPPIV is a cell surface glycoprotein found on cells of the intestinal epithelium including those of the colon. We have previously shown that the dietary flavone apigenin (4′,5,7‐trihydroxyflavone) upregulates CD26/DPPIV on colon cells. Flavonoids such as apigenin interfere with the action of multiple cellular protein kinases and have the capacity to modulate the cell exterior and its ability to interface with the local environment through different signaling pathways. We show here that the ability of apigenin to upregulate CD26/DPPIV is exerted through and requires the activity of casein kinase 2 (CK2). Inhibitors of CK2 that are distinct from apigenin (emodin, 6‐methyl‐1,3,8‐trihydroxyanthraquinone; TBB, 4,5,6,7‐tetrabromobenzotriazole; and DRB, 5,6‐dichlorobenzimidazole 1‐β‐D‐ribofuranoside) showed a dose‐dependent ability to increase CD26/DPPIV and had the same maximal effect when combined with apigenin at submaximal concentrations. Knockdown of CK2 with siRNA abrogated the ability of apigenin to upregulate CD26/DPPIV. Apigenin treatment of cells had no effect on the levels of CK2 protein, consistent with an inhibition of activity of the enzyme. Apigenin's upregulation of CD26/DPPIV in differentiated human colon epithelial cells depends upon inhibition of CK2 activity. This is a key step in enabling apigenin's ability to regulate the functions of intestinal epithelial cells.

## Graphical abstract

The dietary flavone apigenin (4′,5,7‐trihydroxyflavone) upregulates CD26/DPPIV on intestinal epithelial cells. CD26/DPPIV is a cell surface protein that cleaves N‐terminal dipeptides from several key cellular regulators and modulates their activity. A key early step in this action of apigenin is to inhibit the activity of the enzyme casein kinase 2 (CK2). This is a necessary part of apigenin's ability to regulate the multiple functions of CD26/DPPIV on intestinal epithelial cells.

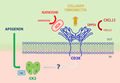

## 1 INTRODUCTION

Apigenin (4′,5,7‐trihydroxyflavone) is a flavone present in the leafy herb parsley and in the dried flowers of chamomile, among other sources. Apigenin has a broad spectrum of activities that affect a variety of cellular processes, as we have reviewed (Lefort & Blay, 2013). These actions endow apigenin with the capacity to alter the relationship of cells with their surroundings and their interaction with key signaling molecules. We have reported that apigenin has the capacity to upregulate CD26/DPPIV (Lefort & Blay, 2011), a key enabler of such interactions with the external milieu (Cheng, Abdel‐Ghany, & Pauli, 2003; De Meester, Korom, Van Damme, & Scharpe, 1999; Dinjens et al., 1986; Dong et al., 1997; Havre et al., 2008; Loster, Zeilinger, Schuppan, & Reutter, 1995; Schrader, West, Miczek, & Norton, 1990). Apigenin was more effective than other related flavonoids in terms of this ability (Lefort & Blay, 2011).

Cluster of differentiation marker 26 (CD26), alternatively known as dipeptidyl peptidase 4 (DPPIV) or adenosine deaminase complexing protein 2, is a 110‐kDa membrane glycoprotein that in its dimeric form is enzymatically active at the cell surface (De Meester et al., 1992; Gorrell, Gysbers, & McCaughan, 2001) and cleaves particular extracellular substrates. CD26/DPPIV is expressed on various cell types, but is most abundant in the epithelium of tissues such as those of the human intestine (Balis, 1985; Kotackova, Balaziova, & Sedo, 2009). Expression levels of intestinal epithelial CD26/DPPIV are known to correspond to cell differentiation, such that differentiated cells at the mucosal surface express higher CD26/DPPIV levels than their progenitors in the mucosal crypts (Darmoul et al., 1991; Dinjens et al., 1989).

The DPPIV enzyme has an intrinsic hydrolase activity that cleaves N‐terminal dipeptides from important extracellular regulatory peptides (De Meester et al., 1999; Havre et al., 2008). This allows sophisticated biological regulation of key cell mediators such as chemokines (Lefort & Blay, 2013). DPPIV has its highest affinity for the chemokine CXCL12 (stromal‐derived factor‐1, SDF‐1), which is the ligand for CXCR4 and CXCR7, two chemokine receptors implicated in cell survival and migration (Luker et al., 2012; Salmaggi et al., 2009). CD26/DPPIV is, however, a trifunctional protein and also (a) acts as the major cellular binding protein for adenosine deaminase, a soluble ecto‐enzyme that catalyzes the hydrolytic deamination of the immunosuppressive metabolite adenosine (Dinjens et al., 1986; Dong et al., 1997; Schrader et al., 1990), and (b) is able to facilitate cellular interactions with the extracellular matrix proteins collagen and fibronectin (Cheng et al., 2003; Loster et al., 1995).

Flavonoids such as apigenin interfere with the activities of a range of cellular protein kinases, so that apigenin might alter CD26/DPPIV and the cell's ability to interface with the local environment through any of multiple signaling pathways. One known target for apigenin's action is casein kinase 2 (CK2, formerly known as casein kinase II). CK2 is a ubiquitous eukaryotic Ser/Thr‐ protein kinase that has more than 200 known targets for phosphorylation in the cell and plays roles in cell proliferation, cell viability, modulation of DNA damage, and control of circadian processes (Allada & Meissner, 2005; Morales & Carpenter, 2004; Pinna & Meggio, 1997; Sarno et al., 2002). CK2 is a tetrameric protein complex, composed of two catalytic subunits (α 42 kDa or α’ 38 kDa) and two regulatory subunits (β 28 kDa) in various combinations (Ahmad, Wang, Unger, Slaton, & Ahmed, 2008; Litchfield, 2003). CK2 is widely distributed in different cellular compartments and therefore able to influence multiple cellular processes and molecules (Kim et al., 2018; Litchfield, 2003; Suhas et al., 2018). We show here that apigenin's ability to upregulate CD26/DPPIV requires inhibition of this key cellular kinase casein, CK2.

## 2 MATERIALS AND METHODS

### 2.1 Culture of human colorectal cells

HT‐29, Caco‐2, HRT‐18, HCT 116, SW480, and SW620 human colorectal cells were obtained from the American Type Culture Collection (ATCC) and were maintained at 37°C in a humidified atmosphere of 90% air/10% CO_2_. The cell lines used have been confirmed for genotype, and cultures regularly tested for mycoplasma using a PCR‐based approach were negative for contamination. All responses have been confirmed in cells within 4 passages of receipt from ATCC.

Cell stocks were cultured in 80‐cm^2^ flasks (Corning), containing Dulbecco's modified Eagle medium (DMEM), supplemented with heat‐inactivated newborn calf serum (NCS; Life Technologies) and passaged with brief exposure to TrypLE™ Express (Life Technologies). For experimental purposes, cells were seeded at a concentration of 90,000 cells/ml unless otherwise indicated. Once cultures reached 60%–70% of confluent density, they were treated with compounds of interest or with control vehicle, as specified in figure legends.

Bovine serum albumin (BSA), dimethyl sulfoxide (DMSO), apigenin, luteolin, genistein, kaempferol, emodin (6‐methyl‐1,3,8‐trihydroxyanthraquinone), TBB (4,5,6,7‐tetrabromobenzotriazole), and DRB (5,6‐dichlorobenzimidazole 1‐β‐D‐ribofuranoside) were obtained from Sigma‐Aldrich. Flavonoids and CK2 inhibitors were dissolved in DMSO to make stock solutions and subsequently further diluted in medium to give a final DMSO concentration of <0.02% (v/v), a concentration that does not affect CD26/DPPIV levels (Tan, Mujoomdar, & Blay, 2004).

### 2.2 Radioimmunoassay for Cellular CD26/DPPIV

Following a 24‐ to 48‐hr treatment as noted, cell surface CD26/DPPIV was quantified using a radioimmunoassay as previously described (Tan et al., 2004). All washes and incubations were performed at 4°C. Wells from a 48 (or 24‐)‐well plate were washed once with 500 µl (750 µl for 24‐well plate) of ice‐cold phosphate‐buffered saline (PBS; 137 mM NaCl, 24.8 mM Tris‐HCl, 5 mM KCL, 0.7 mM Na_2_HPO_4_, 0.5 mM MgSO_4_, and 1 mM CaCl_2_; pH 7.2) containing 0.2% (w/v) BSA and incubated for 1 hr with 125 µl (200 µl for a 24‐well plate) of PBS containing 1% (w/v) BSA and 1 µg/ml mouse anti‐human CD26 (clone M‐A261) monoclonal antibody (mAb) or mouse IgG isotype‐matched control mAb (clone W3/25). Following the incubation period, cells were washed twice with 500 µl (750 µl for a 24‐well plate) of PBS containing 0.2% (w/v) BSA and were incubated for 1 hr with 125 µl (200 µl for a 24‐well plate) of PBS containing 1% (w/v) BSA and 1 µCi/ml ^125^I‐labeled goat anti‐mouse IgG mAb obtained from (PerkinElmer Life Sciences, NEN). Cells received two final washes with 500 µl (750 µl for a 24‐well plate) of PBS containing 0.2% (^w^/_v_) BSA. Finally, 500 µl of 0.5 M NaOH was added to each well in order to solubilize the cells, and radioactivity was assessed using a gamma counter (Model 1,480 Wizard™ 3, Wallac Co.). Radioactive counts were corrected for both nonspecific binding relative to an isotype control and the number of viable cells as assessed by a Coulter^®^ Model ZM151183 particle counter (Beckman Coulter). For CD26/DPPIV quantification including accessible intracellular pools, HT‐29 cells were first rinsed with ice‐cold PBS and permeabilized/ stabilized for 10 min with 500 µl 4% formaldehyde. The cell monolayers were then rinsed with ice‐cold PBS and washed with PBS containing 0.2% (w/v) BSA, prior to assay.

### 2.3 Western blot analysis for CD26/CK2

HT‐29 cells were seeded into 6‐well plates and were grown to 60%–70% confluency, after which they were exposed to apigenin over a time period of 0–60 hr. Cells were washed twice with ice‐cold PBS and dissolved in lysis buffer (50 mM Tris‐HCl, pH 7.4, 1% Nonidet P‐40, 0.25% sodium deoxycholate, 150 mM NaCl) supplemented with 1 mM EDTA, 1 mM NaF, 1 mM phenylmethylsulfonyl fluoride, and 1× protease inhibitor cocktail set 1 (EMD Canada Inc.). Samples were incubated on a plate rotator for 20 min at 4°C and cell lysates then clarified by centrifugation (12,000 × *g* for 20 min). Cellular protein was quantified using Bio‐Rad Protein Assay Dye Reagent (Bio‐Rad Laboratories Inc.). Samples were denatured in Laemmli buffer at 95°C for 5 min, and 15 µg of protein per lane was separated by SDS‐PAGE using 4% stacking and 10% resolving gels as previously described (Tan, Richard, Zhang, Hoskin, & Blay, 2006). Gels were then electroblotted onto a nitrocellulose membrane, blocked with 3% BSA (CD26) or 5% skim milk (CK2), and probed overnight at 4°C with IgG rabbit anti‐human CD26 primary antibody (ab129060; Abcam plc) or IgG mouse anti‐human casein kinase IIα/α′ (clone 31; BD Pharmingen). Membranes were washed 5 times with Tris‐buffered saline with 0.1% Tween‐20 and then incubated with either horseradish peroxidase (HRP)‐conjugated IgG goat anti‐rabbit secondary antibody (Thermo Fisher Scientific) or HRP‐conjugated IgG goat anti‐mouse secondary mAb (Cedarlane Laboratories Ltd.) for 1 hr at room temperature. Protein expression was detected using an enhanced chemiluminescence detection system (Thermo Scientific). To confirm equal protein loading in each sample, the membrane was reprobed either with an IgG rabbit anti‐human α‐tubulin (11H10) primary Ab (Cell Signaling Technology^®^), followed by an HRP‐conjugated IgG goat anti‐rabbit secondary mAb (BD Pharmingen), or with HRP‐conjugated IgG mouse anti‐β‐actin antibody (Abcam plc), as noted.

### 2.4 Silencing of CK2 using siRNA transfection

Small‐interfering RNA (siRNA) transfection of HT‐29 cells was carried out according to the manufacturer's instructions for reverse transfection, using 3.0 μl/well siPORT™ *Amine* Transfection Agent (AM4503) in 24‐well plates. *Silencer^®^* Select validated negative control #1 siRNA was used as a negative control. Cells were seeded in 10% NCS DMEM at a density of 240,000 cells/ml and were transfected with an optimized concentration of 7.5 nM, using 4 validated *Silencer* ^®^ Select siRNA constructs specific for CK2 at 24 hr following seeding, with a medium change in order to reduce cellular cytotoxicity. 36 hr following siRNA transfection for CK2 (when the knockdown became established as evaluated through Western blot), cells were treated with 60 µM apigenin or its equivalent DMSO control. To examine the effect of apigenin on CK2‐knockdown cells, cell surface CD26/DPPIV levels were assessed through a radioimmunoassay 48 hr following treatment; this corresponded to 84 hr after transfection.

### 2.5 Statistical analysis

Unless otherwise noted, figures are representative of at least 3 independent experiments conducted on separate occasions. Statistical analyses were performed using Prism 8.0 software (GraphPad). Comparisons of data were performed using two‐way ANOVA with Bonferroni's comparison test to compare the replicate means unless otherwise indicated. For all analyses, a *p*‐value < .05 was considered as the minimum for statistical significance.

## 3 RESULTS AND DISCUSSION

### 3.1 Apigenin upregulates CD26/DPPIV in colorectal cell lines showing a more differentiated phenotype

**FIGURE 1:**
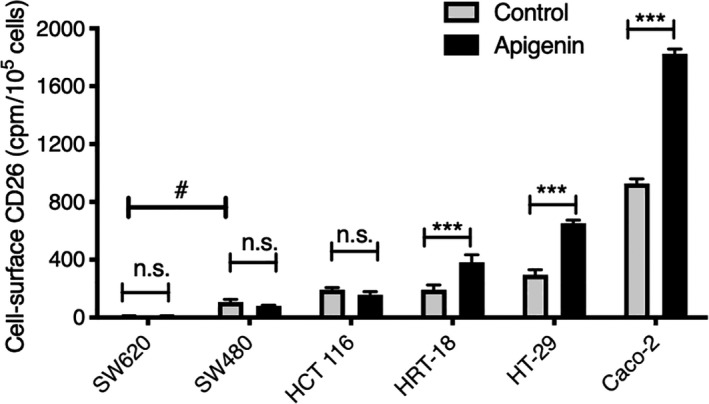
Apigenin upregulation of CD26/DPPIV in different sources of human colon epithelial cells. Cells were treated for 48 hr in the absence or presence of apigenin (30 µM) and cell surface CD26/DPPIV then measured by radioimmunoassay. Data are corrected for nonspecific binding of anti‐CD26 mAb and expressed based on the number of viable cells. Values are means ± *SEM* (*n* = 4); ***, *p* < .001, increase over respective control; n.s., not significant; #, *p* < .05, difference between SW620 and SW480 cell lines

We previously documented the ability of apigenin to upregulate the cell surface expression of CD26/DPPIV on HT‐29 and HRT‐18 human colorectal cells (Lefort & Blay, 2011). The increase in CD26/DPPIV reached a maximum after 24–48 hr, with a mean elevation of 56.3% in response to apigenin, with an EC_50_ of 3‐30 μM (Lefort & Blay, 2011). We examined this response in a broader range of human colon epithelial cells that have different degrees of differentiation (Lea, Ibeh, Han, & Desbordes, 2010; Schneider et al., 2012). In addition to HRT‐18 and HT‐29 cells, well‐differentiated Caco‐2 cells also showed a robust elevation in CD26/DPPIV in response to apigenin, which was an approximate doubling of CD26/DPPIV (Figure 1). In contrast, SW620, SW480, and HCT116 cells showed no CD26/DPPIV upregulation due to apigenin (Figure 1).

The sensitivity to apigenin correlates with a higher initial level of cell surface CD26/DPPIV, although it occurs over a ~4‐fold range of basal CD26/DPPIV levels (HRT‐18 relative to Caco‐2 cells). However, of two cell lines with modest basal CD26/DPPIV levels (HCT116 and HRT‐18) only the latter was responsive to apigenin, indicating that it is not solely the basal CD26/DPPIV level per cell that itself determines responsiveness. Nevertheless, these cell lines are ordered according to their degree of differentiation (Figure 1) and CD26/DPPIV is itself a differentiation marker (Darmoul et al., 1991; Siavoshian et al., 1997; Ten Kate, Wijnen, Boldewijn, Khan, & Bosman, 1985), so it is most likely that the sensitivity to apigenin requires the presence of pathways that are acquired in the later stages of cell differentiation.

### 3.2 Apigenin upregulates CD26 predominantly at the cell surface

We investigated whether the modulation of CD26/DPPIV by apigenin was just at the cell surface, which is the pool measured by our radioimmunoassay on live intact cells, or involved a change in total cellular abundance of CD26/DPPIV.

**FIGURE 2:**
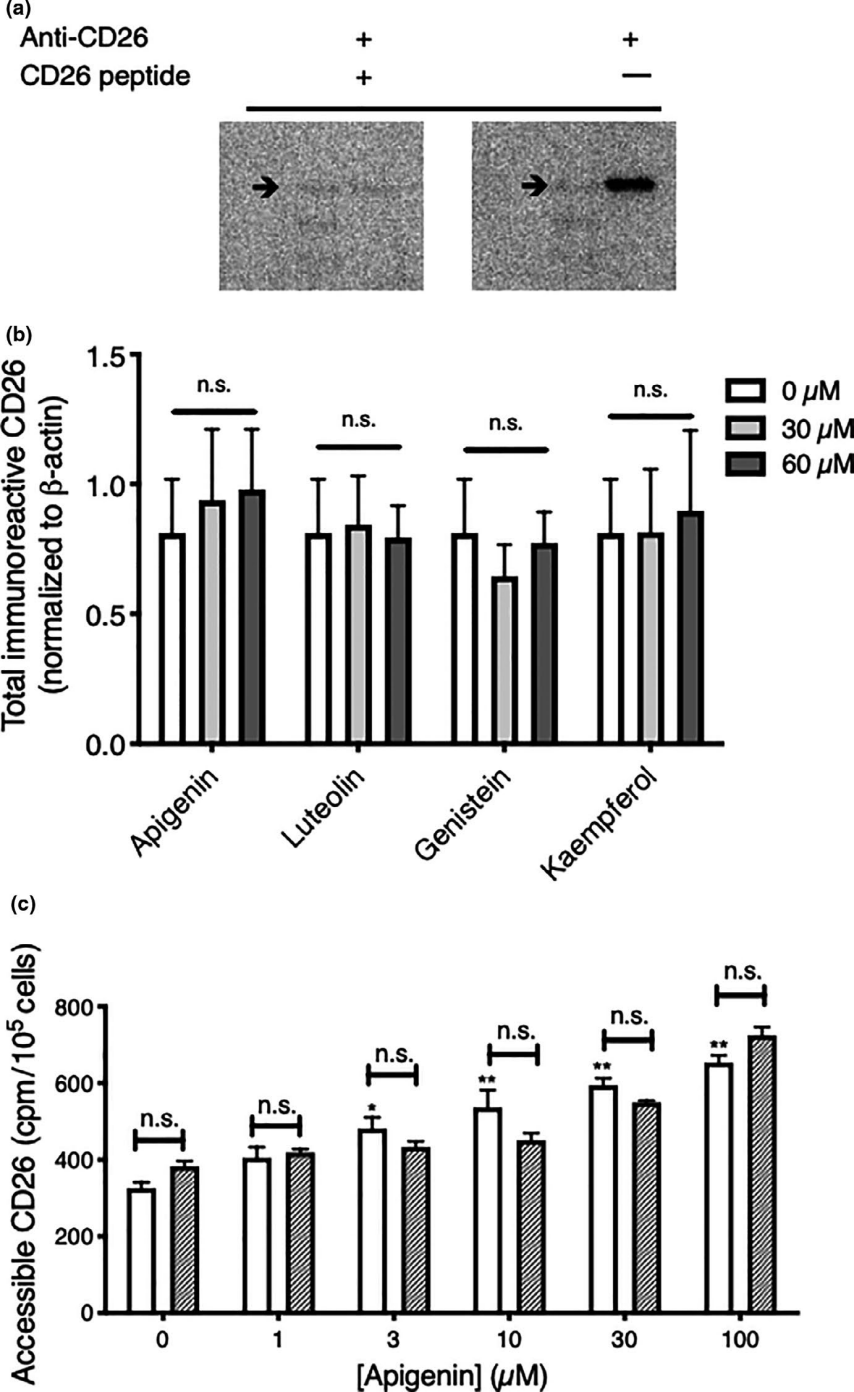
Apigenin does not substantially elevate total cellular CD26/DPPIV or mobilize CD26/DPPIV from accessible intracellular pools. (a) Detection of CD26 protein. Images from Western blotting for purified recombinant CD26 protein using anti‐human CD26 antibody in the absence (−) or presence (+) of neutralizing CD26 peptide. The position of the 100 kDa protein marker is noted (arrow). (b) Apigenin and related flavonoids do not significantly elevate total cellular immunoreactive CD26/DPPIV. HT‐29 cells were treated with flavonoids at the indicated concentrations, solubilized, and the lysates immunoblotted for CD26/DPPIV. Data are mean values ± *SEM* from three independent experiments. n.s., not statistically significant. (c) Apigenin does not recruit CD26/DPPIV to the cell surface from accessible pools. HT‐29 cells were treated in the absence or presence of apigenin and accessible CD26/DPPIV measured by radioimmunoassay in whole cells with plasma membranes intact (clear bars) or cells for which the surface membranes had first been permeabilized (hatched bars). Data are means ± *SEM* (*n* = 4); *, *p* < .05 or **, *p* < .01, increase over control for intact cells; n.s., no significant difference between intact and permeabilized cells

Experiments under a variety of culture conditions failed to reveal a statistically significant increase in total CD26/DPPIV protein due to apigenin in whole cell lysates measured using Western blotting (Figure 2a,b). We also saw no change in total immunoreactive CD26/DPPIV in cell lysates after treatment with the apigenin metabolite luteolin, its molecular isomer genistein, or the hydroxylated derivative kaempferol (Figure 2b). We were also unable to demonstrate upregulation in CD26 mRNA expression due to these flavonoids using quantitative RT‐PCR with normalization to GAPDH (data not shown). The effect of apigenin (and genistein (Lefort & Blay, 2011)) is therefore primarily to redistribute CD26/DPPIV to the cell surface, where mature enzyme has its effects on relevant substrates.

The display of CD26/DPPIV at the cell surface of colorectal cells is highly dependent upon its trafficking between different cellular compartments. The uptake of CD26/DPPIV through endocytosis creates a pool of CD26/DPPIV in cellular vesicles inside the plasma membrane, and CD26/DPPIV may also be stored at other locations within the cell (Klumperman, Boekestijn, Mulder, Fransen, & Ginsel, 1991; Matter, Stieger, Klumperman, Ginsel, & Hauri, 1990). The enhancement of CD26/DPPIV at the cell surface by apigenin might be due to CD26/DPPIV mobilization from easily accessible intracellular pools. However, permeabilization of the plasma membranes of intact cells to include detection of intracellular CD26/DPPIV (Andre‐Garnier et al., 2003; Jalal et al., 1992) did not reveal any accessible internal pools of CD26/DPPIV in HT‐29 cells that contributed to delivery to the cell surface (Figure 2c), suggesting that the CD26 must be delivered from a sequestered pool, perhaps that located at the basolateral membrane of a polarized differentiated epithelial cell (Klumperman et al., 1991; Matter et al., 1990).

### 3.3 Inhibition of CK2 activity results in the elevation of CD26/DPPIV

**FIGURE 3:**
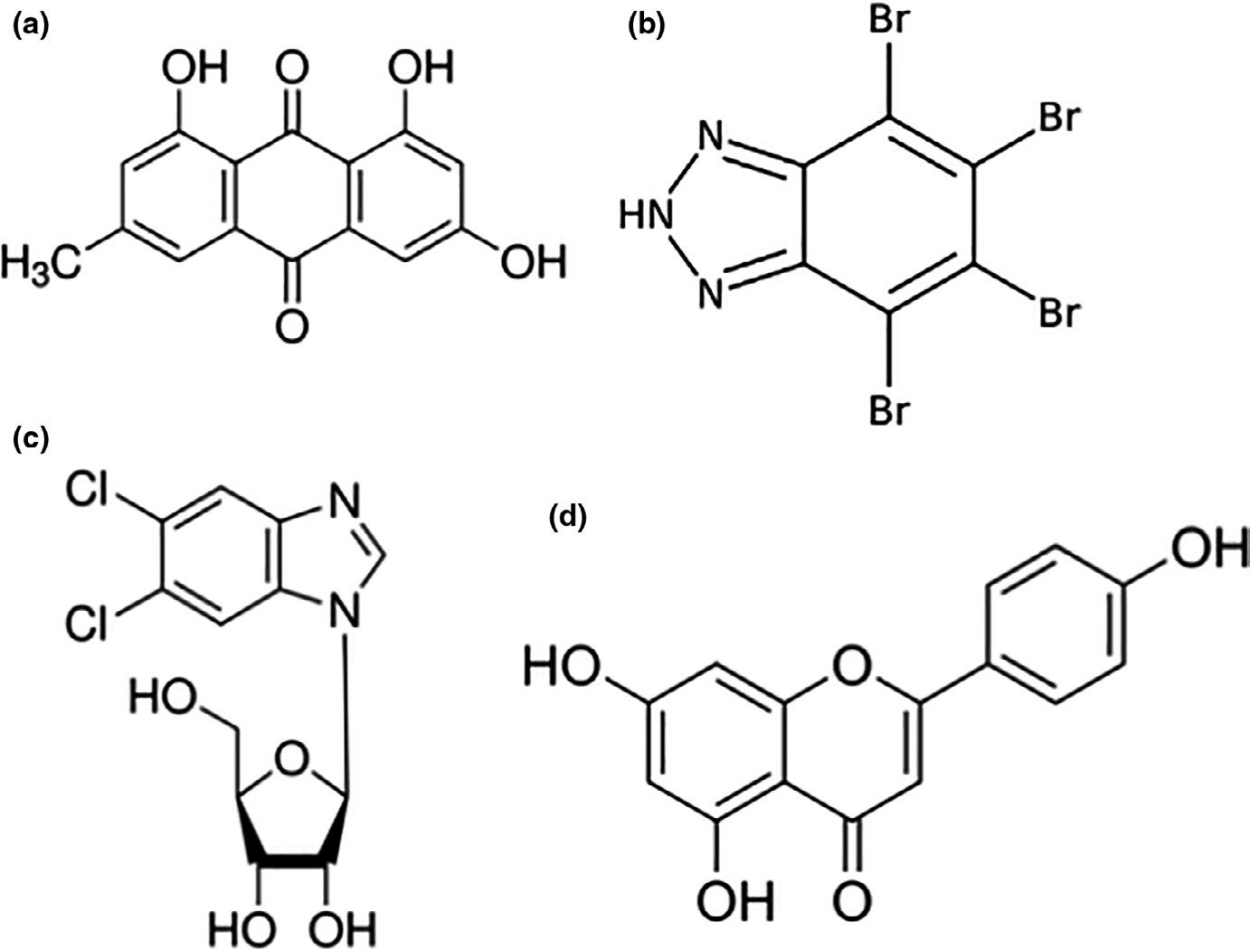
Structures of CK2 inhibitors. (a) Emodin (6‐methyl‐1,3,8‐trihydroxyanthraquinone). (b) TBB (4,5,6,7‐tetrabromo‐2‐azabenzimidazole). (c) DRB (5,6‐dichlorobenzimidazole 1‐β‐D‐ribofuranoside). (d) Apigenin (4′,5,7‐trihydroxyflavone; 5,7‐dihydroxy‐2‐(4‐hydroxyphenyl)‐4H‐1‐benzopyran‐4‐one)

One of the better known properties of apigenin is its ability to inhibit the broad specificity kinase, CK2. If apigenin acts through this route, other CK2 inhibitors should enhance levels of CD26/DPPIV at the cell surface. We tested three CK2 inhibitors for their ability to elevate cell surface CD26: emodin (6‐methyl‐1,3,8‐trihydroxyanthraquinone), TBB (4,5,6,7‐tetrabromo‐2‐azabenzimidazole), and 5,6‐dichlorobenzimidazole 1‐β‐D‐ribofuranoside). The three inhibitors are structurally diverse (Figure 3a‐c) and distinct from apigenin (4′,5,7‐trihydroxyflavone; Figure 3d).

**FIGURE 4:**
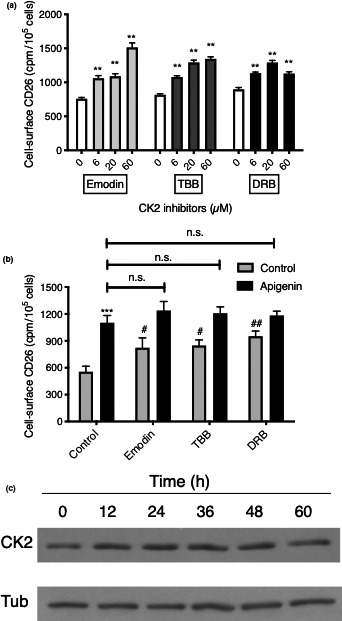
Inhibition of CK2 activity reproduces the apigenin upregulation of CD26/DPPIV. (a) CK2 inhibitors enhance cell surface CD26/DPPIV levels. HT‐29 cells were treated with CK2 inhibitors and cell surface CD26/DPPIV measured after 48 hr. Values are means ± *SEM* (*n* = 4); ANOVA followed by Dunnett's test; **, *p* < .01, significant enhancement over relevant control. (b) CK2 inhibitors do not extend the increase in CD26/DPPIV above the maximum achieved with apigenin. HT‐29 cells were treated with CK2 inhibitors without or with 60 µM apigenin. Concentrations used were the maximum possible without inducing net toxicity and were as follows: emodin 6 µM; TBB 1 µM; DRB 1 µM. Values are means ± *SEM*, 5 independent experiments; ***, *p* < .001, increase due to apigenin; n.s., not significant; #, *p* < .05 or ##, *p* < .01, enhancement by CK2 inhibitors relative to control. (c) Lack of effect of apigenin on CK2 protein levels. HT‐29 cells were treated with apigenin (60 µM) for the times noted and Western‐blotted for CK2 or α‐tubulin (Tub). Representative of *n* = 3 independent experiments

All three distinct CK2 inhibitors, like apigenin, led to an increase in cell surface CD26/DPPIV that was dose‐dependent and maximal in the concentration range 20‐60 µM (Figure 4a). The maximum elevations of CD26/DPPIV in multiple independent experiments were as follows: emodin, 95.5%; TBB, 71.4%, and DRB, 77.4%, compared with apigenin, 98.5% (Figure 4b). Although different molecules, with different activities, noted for other targets (Seifeddine et al., 2008; Song et al., 2013), this shows remarkable congruency in effect on CD26/DPPIV for four very different agents that inhibit CK2.

The addition of CK2 inhibitors did not elevate CD26/DPPIV levels beyond that possible with apigenin alone (Figure 4b). When we added emodin, TBB, or DRB (at the maximum concentrations possible without toxicity) along with a maximally effective concentration (60 µM) of apigenin, there was no increase beyond that of apigenin alone. This suggests that we are dealing with a single pathway through CK2 inhibition.

Given the protracted time course of the apigenin upregulation of CD26/DPPIV activity (maximum reached after 24–48 hr (Lefort & Blay, 2011)), it was likely that apigenin itself was reducing expression of the kinase. However, Western blotting of cell extracts for CK2 protein in apigenin‐treated cells revealed no decline in CK2 over a protracted time course (Figure 4c). The effect of apigenin therefore involves an inhibition of the kinase activity of CK2, in a similar manner to the CK2 inhibitors that reproduce its action.

### 3.4 Knockdown of CK2 abrogates the apigenin elevation of CD26/DPPIV

**FIGURE 5:**
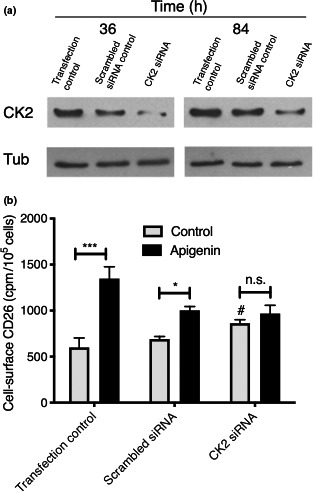
Knockdown of CK2 abrogates the apigenin upregulation of CD26/DPPIV. (a) Knockdown of CK2 levels using an siRNA approach. HT‐29 cells were examined at 36 or 84 hr after transfection as indicated and Western‐blotted for CK2 or α‐tubulin (Tub). Representative of *n* = 3 independent experiments. (b) Knockdown of CK2 abrogates the ability of apigenin to upregulate CD26/DPPIV. Cell surface CD26/DPPIV was evaluated 48 hr following treatment with 60 µM apigenin in the three groups of cells noted in panel A. Means ± *SE* (*n* = 4); *, *p* < .05 or ***, *p* < .001, increase due to apigenin; n.s., not significant; #, *p* < .05, enhancement by CK2 knockdown

If apigenin upregulation of CD26/DPPIV proceeds through CK2 inhibition, it will depend on the presence of functional CK2. In order to decrease the amount of functional CK2, an siRNA approach was employed (Figure 5). Four validated siRNA constructs were used, and the negative (scrambled) control was a validated negative control siRNA from the same manufacturer, used at the same final RNA concentration and ratio with transfection reagent as the CK2‐silencing combination. We were able to achieve a substantial CK2 knockdown that reached 95.4% after 36 hr and was retained at 75.7% 84 hr after the transfection step (Figure 5a, third lanes). We consistently noted a minor but significant reduction in CK2 protein in the presence of the scrambled control siRNA (Figure 5a, second lanes).

Apigenin retained its ability to substantially upregulate (mean increase 125%) CD26/DPPIV in control cells after transfection in spite of the altered seeding and culture conditions necessary for the siRNA approach (Figure 5b). However, the effect of apigenin (60 μM) was eliminated in response to the knockdown of CK2 protein (Figure 5b). CK2 siRNA itself led to a significant increase in CD26/DPPIV relative to the transfection control, as would be expected with loss of its basal kinase activity. For the scrambled siRNA control, mean CD26/DPPIV measures were slightly higher, consistent with its measured small impact on CK2 expression; there was also a reduction in the response to apigenin. With apigenin treatment, the CD26/DPPIV levels were reduced to those of the knockdown control, showing loss of the upregulatory response when CK2 is quantitatively removed as a target option (Figure 5b).

### 3.5 Apigenin upregulation of CD26/DPPIV requires inhibition of CK2

In this work, we have shown that the apigenin upregulation of functional CD26/DPPIV at the cell surface of intestinal epithelial cells (a) is consistent across multiple well‐differentiated human cell lines, (b) is due to cellular redistribution rather than a change in expression, (c) may be reproduced by multiple inhibitors of CK2 kinase activity, (d) does not involve downregulation of CK2 protein, and (e) is abrogated by siRNA knockdown of available CK2. It is well established that apigenin can inhibit CK2 activity, and indeed, it is used experimentally as a CK2 inhibitor (Ahmad, Wang, & Ahmed, 2006; Jung, Kim, & Lee, 2014; Kim et al., 2018; Kroonen et al., 2012; Suhas et al., 2018). We conclude that the ability of apigenin to upregulate CD26/DPPIV involves inhibition of the kinase activity of CK2 and is dependent upon the presence of functional CK2 enzyme.

However, it is not certain that apigenin influences cellular behaviors, including regulation of interactions with the extracellular matrix, cellular contractility, and the levels of molecules including CD26/DPPIV (Kim et al., 2018; Lefort & Blay, 2011; Suhas et al., 2018), solely through inhibition of CK2. The effects of apigenin in cellular experimental systems are distinct from synthetic CK2 inhibitors such as CX‐4945 (Suhas et al., 2018). Furthermore, the CK2 interactions of flavonoid inhibitors such as quercetin and fisetin differ from those of synthetic inhibitors such as emodin and TBB in terms of the residues impacted (Sarno et al., 2002). As well, such interactions differ between flavonoids (Sarno et al., 2002), suggesting that the precise modulation of CK2 activity may vary even between different flavonoids.

### 3.6 Implications for the role of CK2 in the actions of apigenin

Beyond effects on normal intestinal function, dietary apigenin may have its beneficial effects in the context of gastrointestinal cancers, as we have reviewed (Lefort & Blay, 2013), in part due to its ability to inhibit CK2 activity. Casein kinase 2 is elevated in proliferating tissues, both normal and neoplastic, and may contribute to the formation of certain neoplasias (Dubois et al., 2016; Pinna & Meggio, 1997; Sarno et al., 2002). In addition, it may contribute to more aggressive cancer behaviors such as invasion (Kim et al., 2018). There are suggestions that the suppression of CK2 might be a valid therapeutic intervention to reduce the invasive behavior of cancer cells (Kim et al., 2018). However, the protracted time course of the increase in cell surface CD26/DPPIV (24–48 hr) suggests that there are likely significant other events, most probably downstream of CK2, that participate in elevation of CD26/DPPIV. We are currently exploring these possibilities.

In summary, we have shown here that inhibition of CK2 activity is a key proximal step in the ability of apigenin to upregulate CD26/DPPIV in at the cell surface of differentiated human colon epithelial cells.

## CONFLICT OF INTEREST

The authors declare no conflicts of interest.
